# Continuous versus intermittent noninvasive blood pressure measurement in patients with shock in prehospital emergency medicine – a single-center prospective pilot trial

**DOI:** 10.1186/s13049-025-01457-5

**Published:** 2025-08-21

**Authors:** Stephan Katzenschlager, Raphael Heck, Nikolai Kaltschmidt, Frank Weilbacher, Markus A. Weigand, Erik Popp, Maximilian Dietrich

**Affiliations:** Medical Faculty Heidelberg, Department of Anaesthesiology, Heidelberg University, Im Neuenheimer Feld 420, 69120 Heidelberg, Germany

**Keywords:** Emergency medical services, Microcirculation, Blood pressure monitors, Diagnostic techniques and procedures, Shock

## Abstract

**Background:**

Shock is a critical and potentially life-threatening clinical state characterized by circulatory insufficiency and impaired micro- and macrocirculation. Rapid detection and initiation of therapy are essential for patient outcomes. In prehospital emergency medicine, assessment tools are limited, and intermittent noninvasive blood pressure (iNIBP) monitoring is the current standard of care. Recent findings suggest that this method may miss episodes of relevant hypotension. Continuous noninvasive blood pressure (cNIBP) and tissue oxygenation (StO_2_) measurements could improve the time to detection of shock.

**Methods:**

This single-center prospective pilot trial compared a cNIBP system with standard iNIBP measurements in physician-staffed prehospital care. The study was conducted in the Rhine-Neckar region between May and December 2023. The Edwards *HemoSphere* system, including *ClearSight* for cNIBP and *ForeSight* for StO_2_, was used in conjunction with standard monitoring. Adults with shock were eligible for inclusion. Primary endpoint was the agreement between cNIBP and iNIBP; secondary endpoints included unrecognized hypotension (MAP < 60 mmHg) and comparison between cNIBP/iNIBP and StO_2_. Bland-Altman analysis quantified bias and limits of agreement (LoA).

**Results:**

In total, 25 patients were included, resulting in 100 simultaneous measurements. iNIBP readings exceeded cNIBP measurements of mean arterial pressure (MAP) by 10.77 mmHg (*p* < 0.01). There were further significant differences for systolic and diastolic blood pressure, with higher values for iNIBP measurements. Bland-Altman analysis demonstrated systemic bias (MAP bias − 10.25) with wide LoA (-43.52 to 22.21), indicating poor interchangeability. In three out of 25 cases, standard intermittent blood pressure measurements failed to detect hypotension, although cNIBP showed MAP values below 60 mmHg.

**Conclusion:**

Our pilot data show cNIBP and iNIBP values differ significantly, with clinical implications, potentially improving hemodynamic instability detection. However, as this is preliminary, more research on system reliability and benefits of enhanced monitoring is needed.

**Trial registration:**

German Clinical Trials Registry (DRKS ID DRKS00031867) on 22.05.2023.

**Supplementary Information:**

The online version contains supplementary material available at 10.1186/s13049-025-01457-5.

## Introduction

Shock is defined as circulatory failure, characterized by inadequate tissue perfusion and oxygen delivery due to impaired blood flow. If untreated, organ failure and death are the consequences [1].

In early shock, nonspecific signs like elevated respiration and tachycardia indicate a compensatory mechanism to maintain tissue perfusion and oxygen [2].

Prehospital circulation-related problems can develop rapidly, and blood pressure—as a key parameter for assessing circulatory status in shock—is highly dynamic. Intermittent non-invasive blood pressure measurement (iNIBP) carries the risk of missing critically low blood pressure values [3].

The capillary refill time depends on the observer, patient’s age, skin color, temperature, and the applied pressure [4].

Shock index (SI) is defined as the patient’s heart rate and systolic blood pressure index. An SI of > 1.0 is commonly interpreted as a sign of impaired hemodynamic status [5]. Though SI may predict increased morbidity and mortality, confounders such as age, febrile conditions, other illnesses, and medications can alter SI, potentially over- or underestimating hemodynamic impairment [5].

Continuous noninvasive blood pressure (cNIBP) monitoring might fill this gap by providing real-time blood pressure trends, potentially identifying hypotension earlier than intermittent cuffs [6]. Tissue oxygenation saturation (StO_2_) can detect microcirculatory alterations and can demonstrate perfusion changes [7].

A recent study showed that cNIBP reduced the rate of intraoperative hypotension in patients with non-cardiac surgery [8]. Furthermore, a direct comparison of iNIBP and intra-arterial blood pressure measurement (IABP) in the prehospital setting in the UK showed a significant difference between the two methods [9]. In particular, iNIBP values are overestimated for very low and underestimated for very high blood pressure [9]. For adults, physiological limits for mean arterial pressure (MAP) should be 65 mmHg or higher; MAP values below 60 mmHg indicate an impaired macrocirculatory status in otherwise healthy adults [10, 11].

This single-center prospective pilot trial investigates the agreement between cNIBP and iNIBP measurement in a physician-based pre-hospital emergency medicine setting.

## Methods

This single-center, prospective pilot trial was approved by the Ethics Committee of Heidelberg University (S-004/2023) and registered prior to trial enrollment in the German Clinical Trials Registry (DRKS00031867, Registration Date 22.05.2023). The reporting of this trial follows the Strengthening the Reporting of Observational Studies in Epidemiology statement (Supplement STROBE Checklist) [12]. Due to the exploratory character, no formal sample size calculation was performed. We planned to enroll patients over one year.

### Setting

The emergency medical services (EMS) in the Rhine-Neckar region have a two-tier system, with ambulances staffed by one paramedic and one emergency medical technician, and physician response cars staffed by one emergency medicine physician and one paramedic. The rescue control center is responsible for registering every medical emergency call within the region and deciding whether an ambulance is sufficient for the emergency or if a physician response car is required immediately. Moreover, the paramedics can determine whether an emergency physician is needed. To every suspected OHCA or unconscious patient, severe trauma, suspected myocardial infarction, or acute dyspnea, both ambulance and physician response cars are dispatched. Therefore, it captures the subset of patients with circulatory impairment.

Both units have the Corpuls *C3* or Corpuls *C3T* (GS Elektromedizinische Geräte G. Stemple GmbH, Kaufering, Germany) as monitors and defibrillation systems. Some physician response cars have the possibility for IABP monitoring. Standard care involved iNIBP measurements (using the Corpuls device) at intervals determined by the treating team or as clinically needed.

### Enrollment

A study team member randomly accompanied different physician response cars in the Rhine-Neckar EMS system. At the time of data acquisition, the study team member was a medical student and paramedic. Patients over 18 years with shock were eligible for enrollment. Shock could be due to suspected acute coronary syndrome (ACS), trauma, undifferentiated shock, or OHCA. Patients were included if they met the inclusion criteria, and no exclusion criteria were applicable (Table 1).

For the definition of “undifferentiated shock” on the scene, the capillary refill time on the patient’s palm had to be over two seconds or the shock index > 1. “Undifferentiated” referred to shock of any other cause except acute myocardial infarction or trauma.

A suspected ACS was determined by an indicative clinical presentation of patients, with or without specific electrocardiogram findings.

Patients with OHCA were enrolled during ongoing cardiac arrest or return of spontaneous circulation (ROSC), depending on the arrival of the physician response car. After ROSC, measurements were performed until the patient arrived at the hospital.

This patient population was chosen because it reflects the most common and clinically relevant prehospital scenarios associated with hemodynamic instability and the risk of hypotension.

A physician not involved in the study had to determine if the patient’s condition was potentially life-threatening. After positive screening, patients were enrolled without explicit consent under an initial emergency exemption. All patients or their legal representatives were visited or contacted by phone by a study physician after hospital admission to obtain informed consent.

**Table 1:** Inclusion and exclusion criteria

Inclusion criteria	Exclusion criteria
Age ≥ 18 years	Known pregnancy
Capillary refill > 2s or Shock index > 1	Prisoners
ACS or OHCA or trauma with shock or undifferentiated shock	Certain signs of death
	Do not resuscitate order

Abbreviations: ACS = acute coronary syndrome; OHCA = out-of-hospital cardiac arrest

### Study interventions

During the study period, an additional Edwards HemoSphere monitoring system was carried, equipped with the *ClearSight* module for cNIBP and *ForeSight* for StO_2_ measurement via near-infrared spectroscopy (NIRS). If the patient met the inclusion criteria, the study personnel applied the HemoSphere system while the EMS crew established standard monitoring. The ForeSight StO_2_ sensor was placed on the contralateral forearm to the iNIBP cuff. The treating EMS teams were not blinded to the study interventions. The treating teams were allowed to use the cNIBP and StO_2_ values for clinical decision making at their own discretion. After the mission, all monitoring data were extracted.

A detailed description of the HemoSphere system is presented in the supplements (Supplement Text 1, Supplement Figs. 1 and 2).

### Endpoints, data synthesis, and statistical analysis

The primary endpoint was to assess agreement between paired cNIBP and iNIBP measurements. Secondary endpoints included the relationship between StO_2_ and cNIBP and iNIBP measurements. Further, we evaluated the duration of unrecognized hypotension phases. A hypotensive phase was defined as a MAP of 60 mmHg or lower.

Descriptive statistics were performed for continuous parameters using mean and standard deviation (SD) or median and interquartile range (IQR). For categorical parameters, counts and percentages were used.

Continuous parameters were assessed for homogeneity and analyzed using a paired Student’s t-test with a 95% confidence interval (CI) and Pearson correlation with a predefined interpretation (Supplement Table 1) [13]. Agreement between cNIBP and iNIBP measurements was assessed using Bland-Altman analysis [14]. For each parameter, the bias (mean difference) and the 95% limits of agreement (LoA) were calculated. In addition, 95% confidence intervals were computed for the bias and the LoA to quantify the precision of the estimates [15, 16]. Results were visualized as Bland-Altman plots indicating the bias and LoA. Analyses were performed using only complete measurement pairs.

Differences in StO_2_ between the MAP groups of < 60 mmHg and ≥ 60 mmHg were compared using analysis of variance (ANOVA). A two-sided significance level was defined at *p* < 0.05. Only cases with a complete dataset were included. For all analyses, the latest version of SPSS was used.

## Results

**Fig. 1:**
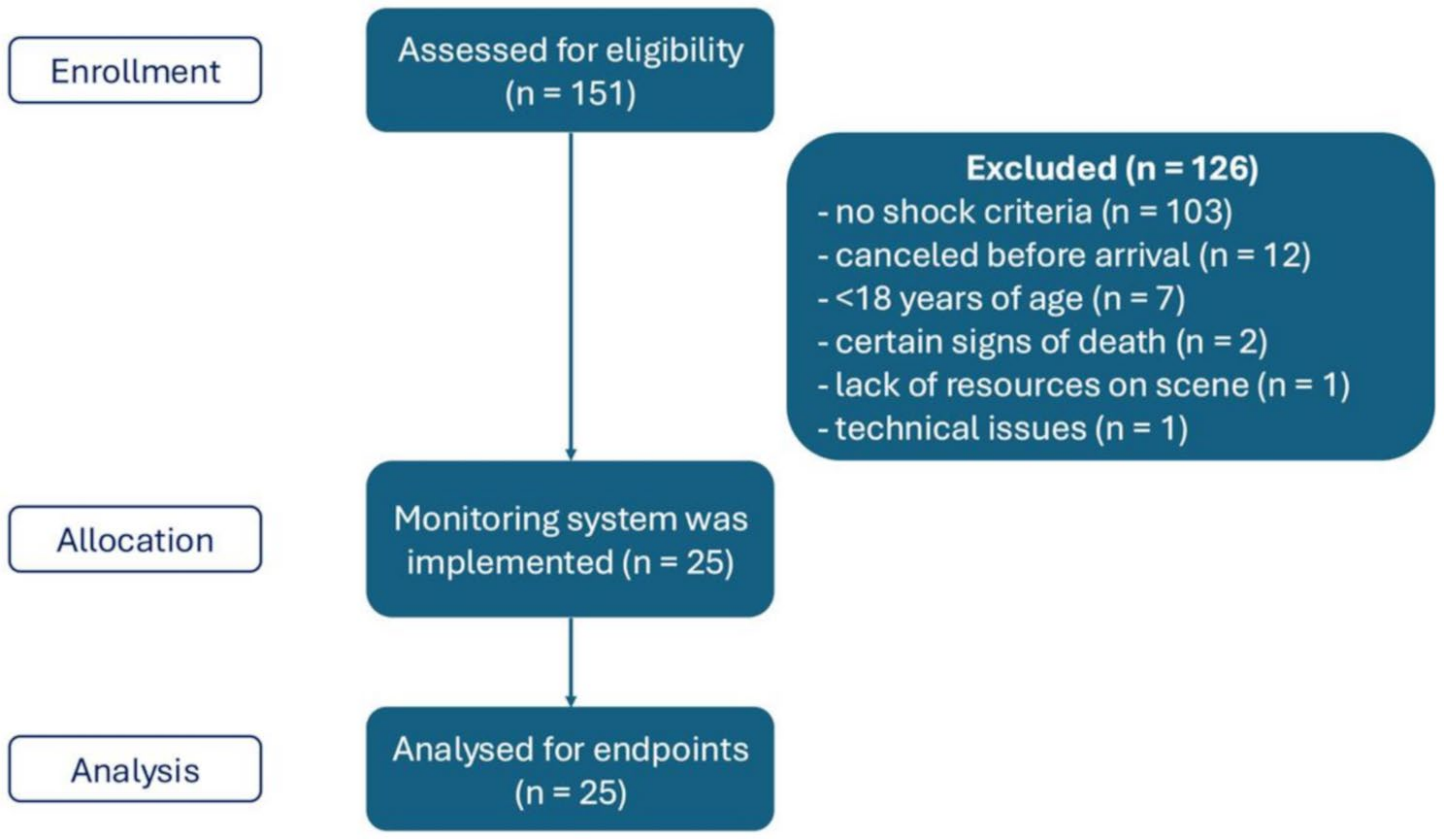
Study flowchart

Between May 30th and December 31st, 2023, 151 cases were screened on a physician response car; of those, 126 patients were excluded after screening. Exclusion was mainly due to the absence of the defined criteria of shock (*n* = 103). In one case, the situation on the scene did not allow the implementation of the study system due to the lack of resources. In another case, there was no possibility to recharge the system between calls, so the *HemoSphere* was out of power and could not be implemented on the patient, despite meeting the inclusion criteria. A total of twenty-five patients were enrolled in the study (Fig. 1). Both the *ClearSight* and *ForeSight* systems could be established in all included cases.

Overall, 25 patients with shock were included in this study. Across all cohorts, 28% were female, and 72% were male. The mean age was 69 years (SD 14 years) (Table 2). The shock cohort included different etiologies (53% cardiac, 27% hypovolemic; Table 2), and 4 out of 15 received vasopressor support in the field. All ACS patients received antiplatelet and, the majority, anticoagulant therapy. One patient did not receive anticoagulation treatment because they were already on oral anticoagulation therapy. In the shock cohort, all patients had a capillary refill time > 2 s, and six patients (40%) had a positive shock index at the arrival of the emergency physician.

**Table 2:** Demographic parameters

Parameter	Overall (*n* = 25)
*Patient characteristics*	
**Age**, mean (SD)*	69 (14)
**Male sex**, ***n*** **(%)**	18 (72%)
*Event details*	
**Location**, ***n*** **(%)**	
Home	15 (60%)
Public place	2 (8%)
Medical facility	8 (32%)
**Presumed cause**, ***n*** **(%)** cardiac anaphylactic hypovolemic infection/sepsis unclear	- 11 (44%) 2 (8%) 4 (16%) 1 (4%) 7 (28%)
**STEMI**, ***n*** **(%)** **NSTEMI**, ***n*** **(%)**	4 (16%) 3 (12%)
**ROSC**, ***n*** **(%)**	2 (8%)
*Prehospital Interventions*	
**i.v. access**, ***n*** **(%)**	25 (100%)
**Medication administered**, ***n*** **(%)** Noradrenaline Adrenaline No vasopressors Aspirin Heparin	3 (12%) 6 (24%) 16 (64%) 7 (28%) 7 (28%)

*p = 0.034 for comparison between ACS and Shock cohort

Abbreviations: ACS: acute coronary syndrome; OHCA: out-of-hospital cardiac arrest; STEMI: ST-elevation myocardial infarction; NSTEMI: non-ST-elevation myocardial infarction; ROSC: return of spontaneous circulation; i.v.: intravenous; SD: standard deviation

### cNIBP vs. iNIBP

Throughout all missions, significant differences were found for systolic, diastolic, and MAP measurements between cNIBP and iNIBP (Table 3).

**Table 3:** Differences between all cNIBP and iNIBP measurements

	cNIBP (*n* = 1511)	iNIBP (*n* = 174)	Mean difference (95% CI)	*p*-value
Systole, mean (SD)	113 (32)	120 (33)	6 (2–12)	0.009
MAP, mean (SD)	83 (22)	91 (23)	8 (4–11)	< 0.01
Diastole, mean (SD)	66 (18)	75 (20)	10 (6–11)	< 0.01

Abbreviations: cNIBP: continuous noninvasive blood pressure; iNIBP: intermittent noninvasive blood pressure; CI: confidence interval; SD: standard deviation; MAP: mean arterial pressure

### Head-to-head comparison cNIBP vs. iNIBP

In total, 100 paired measurements of cNIBP and iNIBP were compared. Mean systolic BP was 129 mmHg by the iNIBP cuff vs. 115 mmHg by cNIBP monitoring, resulting in a difference of 14 mmHg (95% CI 8–19). Similar gaps were observed for diastolic and MAP measurements, with iNIBP averaging a 10 mmHg higher MAP (*p* < 0.01). cNIBP and iNIBP readings showed moderate correlation (Table 4), indicating some agreement in trends.

**Table 4:** Differences and correlation between paired cNIBP and iNIBP

	cNIBP (*n* = 100)	iNIBP (*n* = 100)	Mean difference (95% CI)	*p*-value	Pearson correlation
Systole, mean (SD)	115 (31)	129 (26)	14 (8–19)	< 0.01	0.487
MAP, mean (SD)	85 (20)	96 (18)	11 (8–14)	< 0.01	0.600
Diastole, mean (SD)	69 (17)	79 (16)	10 (7–14)	< 0.01	0.493

Abbreviations: cNIBP: continuous noninvasive blood pressure; iNIBP: intermittent noninvasive blood pressure; CI: confidence interval; SD: standard deviation; MAP: mean arterial pressure

**Fig. 2:**
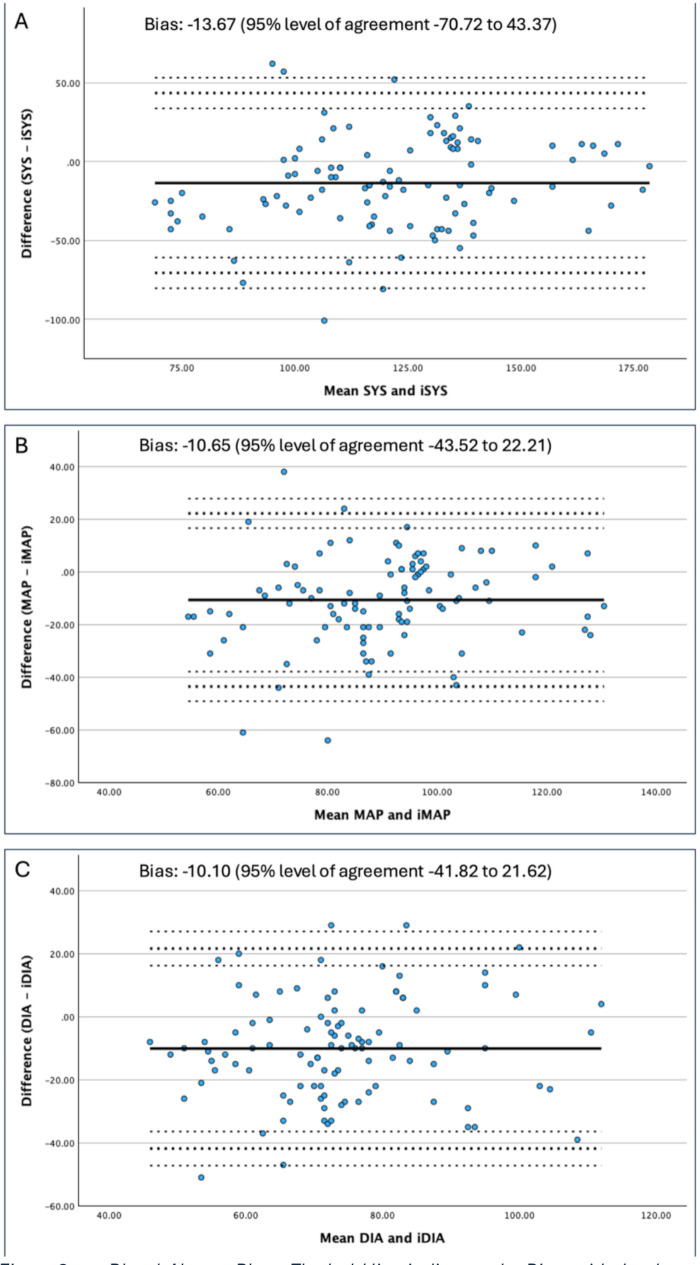
(**a-c**) Bland-Altman Plots. The bold line indicates the Bias, with the dotted line showing the 95% level of agreement and their respective 95% confidence interval. Abbreviations: SYS: continuous systolic blood pressure; iSYS: intermittent systolic blood pressure; MAP: continuous mean arterial pressure; iMAP: intermittent mean arterial pressure; DIA: continuous diastolic blood pressure; iDIA: intermittent diastolic blood pressure

Bland-Altman analysis demonstrated a systematic difference between iNIBP and cNIBP measurements across all parameters. For MAP, the mean bias was − 10.65 mmHg (iNIBP higher), with 95% LoA − 43.52 to 22.21 mmHg. Systolic and diastolic pressures showed similar patterns of bias and wide LoAs, indicating substantial dispersion between the two methods (Fig. 2a-c).

Episodes of hypotension were observed in 3 out of 25 patients that were not captured or measured higher by the corresponding iNIBP readings at those time points (Supplemental Figs. 4a, b,c).

### Tissue oxygenation

The median StO2 values are presented in the Supplement (Supplement Tables 2 and 3; Supplement Fig. 3).

Overall correlation between cNIBP or iNIBP with StO_2_ was poor, with the best correlation for continuous systolic blood pressure (Pearson correlation 0.172) and continuous MAP (Pearson correlation 0.125) (Supplement Table 4).

Simultaneous StO_2_ and cMAP values were compared, with measurements classified as having a cMAP < 60 mmHg (*n* = 189) or ≥ 60 mmHg (*n* = 1230). This resulted in a mean StO_2_ of 68% (95% CI, 66–70) in the < 60 mmHg group and 72% (95% CI, 71–72; *p* < 0.01) in the ≥ 60 mmHg group (Supplement Table 5). When considering measurements for patients in shock, we found higher values for the group ≥ 60mmHg with a mean difference of 1.8% (95% CI 0.5–3.2; *p* = 0.007) (Supplement Table 6).

## Discussion

This pilot trial showed a significant difference between paired cNIBP and iNIBP parameters in the prehospital setting. In the paired comparison (*n* = 100), cNIBP was on average lower than iNIBP for MAP measurements (mean difference 11 mmHg; 95% CI 8–14) and similarly lower for systolic and diastolic pressures, indicating systemic disagreement between the two methods. Bland-Altman analysis corroborated this pattern, implying poor interchangeability at the individual-measurement level and substantial dispersion that could meaningfully alter clinical decision-making.

In some cases, we observed a discrepancy between the cNIBP and iNIBP MAP measurement regarding the clinically significant threshold of 60 mmHg, with higher values in the iNIBP measurements.

Recent findings suggest that iNIBP is inferior to IABP in pre-hospital emergency medicine and to cNIBP in the hospital setting [8, 9], indicating that the accuracy of iNIBP, especially in the pre-hospital setting, warrants closer examination. The iNIBP might overestimate blood pressure in patients with impaired hemodynamic conditions [9]. The higher iNIBP measurements compared to cNIBP observed in this pilot study align with findings reported in the current literature [17].

Clinically, an average MAP gap of 11 mmHg is significant in shock resuscitation, where therapeutic thresholds are narrow. Contemporary guidelines recommend an initial target MAP ≥ 65 mmHg in septic shock and note that perfusion commonly deteriorates below 60 mmHg; delays in achieving ≥ 65 mmHg and in vasopressor initiation are associated with worse outcomes [18]. Requiring precise measurement, especially in the emergency department or prehospital care. In our cohort, cNIBP identified hypotensive episodes (MAP < 60 mmHg) that iNIBP either recorded higher or failed to capture entirely. Illustrating how intermittent measurements may misclassify hemodynamic status and potentially delay escalation [17]. Beyond prehospital care, even brief intraoperative hypotension has been repeatedly linked to myocardial and renal injury, underscoring the plausibility that missed or delayed detection of low MAP translates to harm [19]. While our study was not powered to test patient-centered outcomes, the conjunction of (A) systematic positive bias of iNIBP versus cNIBP, (B) wide limits of agreement, and (C) observed missed hypotension argues that continuous monitoring can reduce false reassurance near actionable thresholds and enable earlier, targeted resuscitation in the field [8, 9].

cNIBP values were displayed on the screen in all cases, although the device failed to record data during movement. On the one side, this could enable clinicians to treat patients with hypotension accurately, which would be missed due to the measurement intervals with iNIBP. On the other hand, non-stored values can lead to documentation issues and can impair comprehensibility. The system could be set up in approximately one minute, with no interference to standard monitoring noted. The impact of *HemoSphere* monitoring data on clinical decision-making was not within the scope of this study and thus was not analyzed separately.

StO_2_ was modestly higher when contemporaneous cMAP was ≥ 60 mmHg; however, correlations between StO2 and both cNIBP and iNIBP were weak. Taken together, these data support StO2 as an adjunct marker of microcirculatory status rather than a surrogate for blood pressure in prehospital shock assessment.

At this stage, cNIBP and StO_2_ would augment, not replace, current monitoring in prehospital emergency medicine.

This study has several limitations. It had to be terminated during the data acquisition as the manufacturer did not extend the loan agreement. With 25 included patients, the trial is significantly underpowered. While we recognize that, our goal was to perform a pragmatic pilot trial to assess the usefulness of cNIBP monitoring across various pathophysiological conditions where rapid changes in circulatory status are anticipated. In addition, in patients with severely impaired circulatory conditions or peripheral arterial disease, the *ClearSight* system sometimes recorded and stored data to a minimal extent. However, pulse wave and cNIBP values were displayed on the monitor. Movements of the patients and the environment, for example, during transportation, often resulted in artefacts and measurement limitations. Previous studies with *ClearSight* were typically conducted on inpatients in intensive care units or during general anesthesia, resulting in significantly fewer movement artifacts.

We did not have invasive arterial pressure measurements to confirm which method (cNIBP or iNIBP) was more accurate; however, given prior evidence of oscillometric inaccuracy in low-pressure ranges, it is plausible that cNIBP provided closer-to-true values in hypotensive patients [20]. The study was planned and conducted as a single-center trial and carried out mainly by one study member accompanying the physician’s response car. This might bias the findings regarding the evaluation of the initial patient status by the study member. The initial diagnosis of shock due to SI or prolonged CRT was not confirmed with in-hospital data; therefore, the initial false-positive definition of shock might have remained undetected. Furthermore, it should be noted that the mean MAP was 85mmHg.

Due to the single-center nature of the study, generalizability is limited, and results might differ in paramedic-only systems or other countries.

Further studies should focus on the missed hypotension phases between cNIBP and iNIBP, as well as the potential to improve hemodynamics. Multi-center studies can statistically confirm the frequency of missed hypotension and evaluate if earlier detection translates to faster interventions or better patient outcomes. Future research should also focus on trauma patients and the increased reliability of the used system.

## Conclusion

Our pilot data indicate that cNIBP and iNIBP values significantly differ with relevant clinical implications. This has the potential to improve the detection of hemodynamic instability. However, given the preliminary nature of this study, further research is needed on subjects such as the reliability of the systems in use and the potential benefits of enhanced monitoring.

## Supplementary Information

Below is the link to the electronic supplementary material.

Supplementary Material 1

## Data Availability

The complete datasets used during the current study are available from the corresponding author on reasonable request.

## Declarations

### Artificial intelligence

To enhance linguistic clarity and precision, the artificial intelligence tool ChatGPT (OpenAI) was utilized to assist with revising and optimizing the manuscript’s language. Its use was limited to language enhancement and structuring, without influencing the scientific content.

## Abbreviations

ACS: Acute coronary syndrome
CI: Confidence interval
cNIBP: Continuous noninvasive blood pressure
EMS: Emergency medical services
iNIBP: Intermittent noninvasive blood pressure
MAP: Mean arterial pressure
NIRS: Near infrared spectroscopy
OHCA: Out-of-hospital cardiac arrest
ROSC: Return of spontaneous circulation
SD: Standard deviation
SI: Shock index
StO_2_: Tissue oxygenation
