# Supplementary material for: Continuous versus intermittent noninvasive blood pressure measurement in patients with shock in prehospital emergency medicine – a single-center prospective pilot trial
Source: Scand J Trauma Resusc Emerg Med. 2025 Aug 21;33:143. doi: 10.1186/s13049-025-01457-5 (PMC12369113; doi:10.1186/s13049-025-01457-5)
Supplement: Supplementary file 1 — Supplementary Material 1 [file 13049_2025_1457_MOESM1_ESM.docx]

**Supplemental Material to**

Continuous versus intermittent noninvasive blood pressure measurement in patients with shock in prehospital emergency medicine – a single-center prospective pilot trial

Stephan Katzenschlager^1^, Raphael Heck^1^, Nikolai Kaltschmidt^1^, Frank Weilbacher^1^, Markus A. Weigand^1^, Erik Popp^1^, Maximilian Dietrich^1,*^

1 Department of Anaesthesiology, Heidelberg University, Medical Faculty Heidelberg, Heidelberg, Germany

* Corresponding Author:

Priv. Doz. Dr. med. Maximilian Dietrich, DESAIC

Heidelberg University, Medical Faculty Heidelberg, Department of Anaesthesiology, Heidelberg, Germany

Im Neuenheimer Feld 420, 69120 Heidelberg, Germany

maximilian.dietrich@med.uni-heidelberg.de

Table of Contents

[Supplement STROBE Checklist 3](#_Toc205804317)

[Supplemental Text 1 – Principles of continuous noninvasive blood pressure measurement (cNIBP) and tissue oxygenation measurement (StO_2_) 6](#_Toc205804318)

[Supplementary Figure 1 – Clinical Setup of the Edwards HemoSphere ClearSight system 7](#_Toc205804319)

[Supplementary Figure 2 – Edwards HemoSphere Monitoring showed continuous blood pressure tracing 8](#_Toc205804320)

[Supplement Table 1 – Pearson correlation definition 9](#_Toc205804321)

[Supplement Table 2- Descriptive statistics and mean difference of StO_2_ in % 10](#_Toc205804322)

[Supplement Table 3 – Mean difference of StO_2_ between subgroups 10](#_Toc205804323)

[Supplement Table 4 - StO2 comparison with continuous and intermittent blood pressure measurements. 10](#_Toc205804324)

[Supplement Table 5 - StO_2_ comparison in groups cMAP < 60mmHg and ≥ 60 mmHg for all patients 10](#_Toc205804325)

[Supplement Table 6 - StO_2_ comparison in groups cMAP < 60mmHg and ≥ 60 mmHg for patients in the “shock” group 11](#_Toc205804326)

[Supplemental Figure 3 – Box Plots for StO_2_ comparisons 12](#_Toc205804327)

[Supplemental Figure 4 – Missed hypotension (MAP ≤60mmHg) 13](#_Toc205804328)

[References 14](#_Toc205804329)

# Supplement STROBE Checklist

|  | Item No | Recommendation | Page No |
| --- | --- | --- | --- |
| **Title and abstract** | 1 | (*a*) Indicate the study’s design with a commonly used term in the title or the abstract | Titel  Abstract, Methods |
|  |  | (*b*) Provide in the abstract an informative and balanced summary of what was done and what was found | Abstract |
| Introduction | | | |
| Background/rationale | 2 | Explain the scientific background and rationale for the investigation being reported | Introduction |
| Objectives | 3 | State specific objectives, including any prespecified hypotheses | Introduction, last paragraph |
| Methods | | | |
| Study design | 4 | Present key elements of study design early in the paper | Methods, first paragraph |
| Setting | 5 | Describe the setting, locations, and relevant dates, including periods of recruitment, exposure, follow-up, and data collection | Methods, Setting |
| Participants | 6 | (*a*) Give the eligibility criteria, and the sources and methods of selection of participants. Describe methods of follow-up | Methods, Enrollment, Table 1 |
|  |  | (*b*) For matched studies, give matching criteria and number of exposed and unexposed | N/A |
| Variables | 7 | Clearly define all outcomes, exposures, predictors, potential confounders, and effect modifiers. Give diagnostic criteria, if applicable | Methods, Endpoints, Data synthesis, and statistical analysis |
| Data sources/ measurement | 8* | For each variable of interest, give sources of data and details of methods of assessment (measurement). Describe comparability of assessment methods if there is more than one group | Methods, Endpoints, Data synthesis, and statistical analysis |
| Bias | 9 | Describe any efforts to address potential sources of bias | Methods, Endpoints, Data synthesis, and statistical analysis |
| Study size | 10 | Explain how the study size was arrived at | Methods, Endpoints, Data synthesis, and statistical analysis |
| Quantitative variables | 11 | Explain how quantitative variables were handled in the analyses. If applicable, describe which groupings were chosen and why | Methods, Endpoints, Data synthesis, and statistical analysis |
| Statistical methods | 12 | (*a*) Describe all statistical methods, including those used to control for confounding | Methods, Endpoints, Data synthesis, and statistical analysis |
|  |  | (*b*) Describe any methods used to examine subgroups and interactions | Methods, Endpoints, Data synthesis, and statistical analysis |
|  |  | (*c*) Explain how missing data were addressed | Methods, Endpoints, Data synthesis, and statistical analysis |
|  |  | (*d*) If applicable, explain how loss to follow-up was addressed | N/A |
|  |  | (*e*) Describe any sensitivity analyses | N/A |
| Results | | |  |
| Participants | 13* | (a) Report numbers of individuals at each stage of study—eg numbers potentially eligible, examined for eligibility, confirmed eligible, included in the study, completing follow-up, and analysed | Results, Figure 1 |
|  |  | (b) Give reasons for non-participation at each stage | Results, Figure 1 |
|  |  | (c) Consider use of a flow diagram | Figure 1 |
| Descriptive data | 14* | (a) Give characteristics of study participants (eg demographic, clinical, social) and information on exposures and potential confounders | Results, Table 2 |
|  |  | (b) Indicate number of participants with missing data for each variable of interest | N/A |
|  |  | (c) Summarise follow-up time (eg, average and total amount) | N/A |
| Outcome data | 15* | Report numbers of outcome events or summary measures over time | Results, cNIBP vs. iNIBP, Table 3 |

| Main results | 16 | (*a*) Give unadjusted estimates and, if applicable, confounder-adjusted estimates and their precision (eg, 95% confidence interval). Make clear which confounders were adjusted for and why they were included | Results, Table 3 |
| --- | --- | --- | --- |
|  |  | (*b*) Report category boundaries when continuous variables were categorized | N/A |
|  |  | (*c*) If relevant, consider translating estimates of relative risk into absolute risk for a meaningful time period | N/A |
| Other analyses | 17 | Report other analyses done—eg analyses of subgroups and interactions, and sensitivity analyses | Results, Figure 2, Supplement Table 2, 3, 4 |
| Discussion | | | |
| Key results | 18 | Summarise key results with reference to study objectives | Discussion, first paragraph |
| Limitations | 19 | Discuss limitations of the study, taking into account sources of potential bias or imprecision. Discuss both direction and magnitude of any potential bias | Discussion, last paragraph |
| Interpretation | 20 | Give a cautious overall interpretation of results considering objectives, limitations, multiplicity of analyses, results from similar studies, and other relevant evidence | Conclusion |
| Generalisability | 21 | Discuss the generalisability (external validity) of the study results | Discussion |
| Other information | | | |
| Funding | 22 | Give the source of funding and the role of the funders for the present study and, if applicable, for the original study on which the present article is based | Declarations, Funding |

# Supplemental Text 1 – Principles of continuous noninvasive blood pressure measurement (cNIBP) and tissue oxygenation measurement (StO_2_)

The cNIBP of the *ClearSight* systems is based on the “volume clamp” method, first described by J. Penaz in 1973. Using a finger cuff with a light source and detector integrated into the cuff, the arterial wall is maintained at a specific diameter through real-time pressure adjustments within the finger cuff. The arterial wall is unloaded, and the transmural pressure difference is zero. Therefore, pressure changes in the cuff correspond to the intra-arterial pressure [1]. The pressure fluctuations triggered by pulse waves and the necessary adjustments of the cuff pressure are converted into pulse pressure by the device and displayed on the monitor in real-time [2].

In contrast to IABP, the *ClearSight* system is entirely noninvasive and can be established quickly. The device does not need an extra iNIBP for pressure calibration. Instead, a reference sensor is placed at the heart's level, like conventional intra-arterial blood pressure (IABP) systems. The device requires approximately 20 seconds of continuous, uninterrupted measurement to save parameters to its internal memory. Thus, three cNIBP measurement points per minute are stored in the internal memory. The *ClearSight* system was validated against iNIBP and IABP in the approval studies [3], [4].

The *ForeSight* tissue oxygenation sensor, which is part of the same monitoring system, is based on near-infrared spectroscopy (NIRS). The detailed NIRS methodology has been described in other trials [5, 6]. In simple terms, a light source emits light at defined wavelengths into the tissue, which is then received by a sensor. Oxyhemoglobin and deoxyhemoglobin absorb light at different wavelengths, which the sensor can detect [6]. The *ForeSight* sensor uses a LED light source that emits light at five defined wavelengths as well as two detectors one 1,5cm and one 5cm away from the light source. The determined tissue oxygenation is displayed as an absolute oxygenation parameter on the display. Validation has been performed for cerebral and somatic tissue oxygenation[6]. Mean values over 20 seconds are saved on the internal memory.

The *HemoSphere* system itself is powered via a 230V power supply for stationary use. Additionally, an internal accumulator provides power for approximately 25-30 minutes of mobile use. During calls with an on-scene time of over 30 minutes, the battery was replaced on-scene, resulting in short measurement interruptions. During transport, the accumulator could be recharged via the ambulance’s electric power supply.

# Supplementary Figure 1 – Clinical Setup of the Edwards HemoSphere ClearSight system


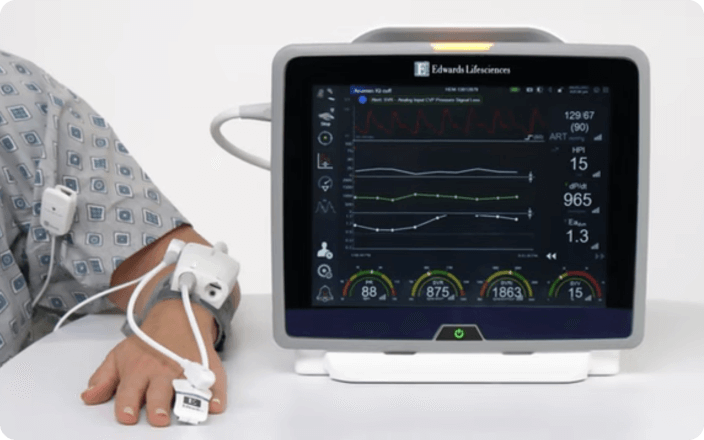


Source: <https://www.edwards.com/healthcare-professionals/products-services/predictive-monitoring/acumen-iq-cuff>, accessed on 11. August 2025

# Supplementary Figure 2 – Edwards HemoSphere Monitoring showed continuous blood pressure tracing


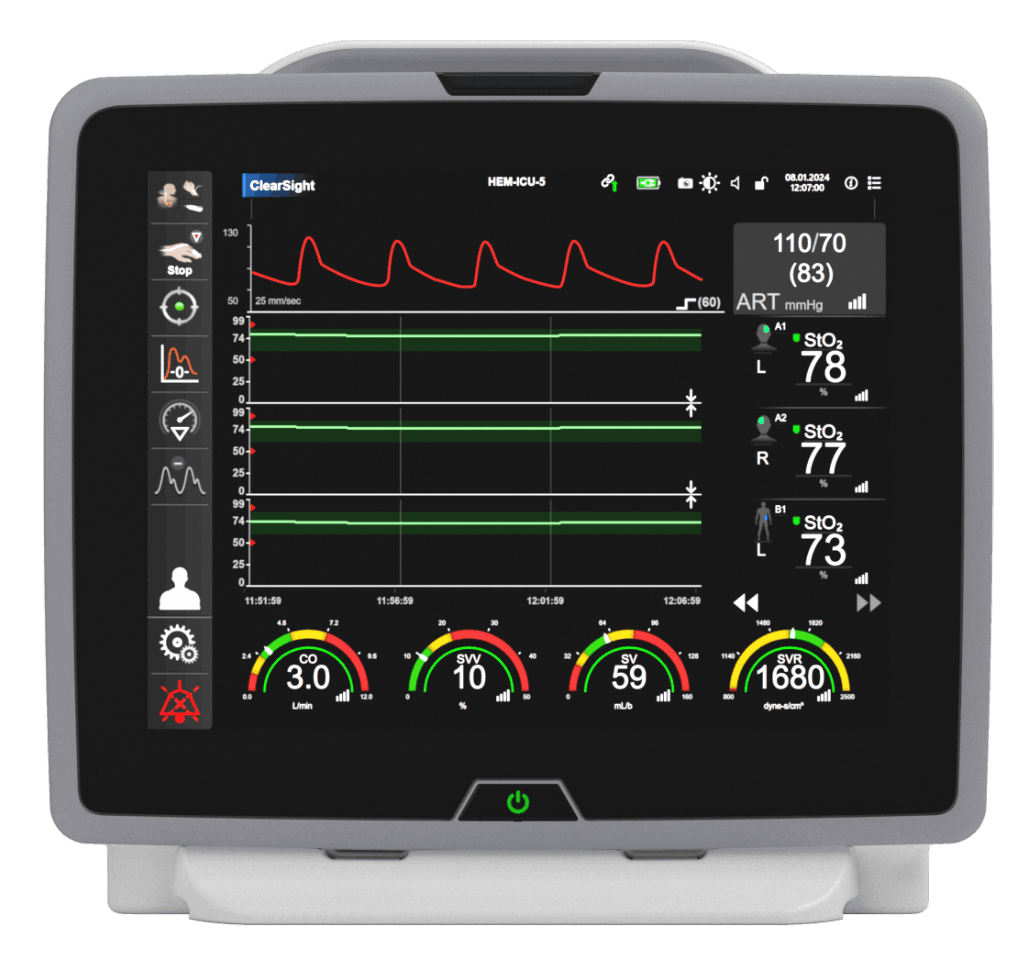


Source: <https://www.edwards.com/healthcare-professionals/products-services/predictive-monitoring/acumen-iq-cuff>, accessed on 11. August 2025

# Supplement Table 1 – Pearson correlation definition

| **Absolute magnitude of observed correlation coefficient** | **Interpretation** |
| --- | --- |
| 0.00 – 0.10 | negligible correlation |
| 0.10 – 0.39 | weak correlation |
| 0.40 – 0.69 | moderate correlation |
| 0.70 – 0.89 | strong correlation |
| 0.90 – 1.00 | very strong correlation |

According to  [7]

# Supplement Table 2- Descriptive statistics and mean difference of StO_2_ in %

|  | Mean | SD | Minimum | maximum |
| --- | --- | --- | --- | --- |
| Shock | 69.1 | 7.6 | 39 | 91 |
| ACS | 72.8 | 8.6 | 41 | 98 |
| OHCA | 62.9 | 15.1 | 28 | 90 |

*Abbreviations: SD: standard deviation; ACS: acute coronary syndrome; OHCA: out-of-hospital cardiac arrest*

# Supplement Table 3 – Mean difference of StO_2_ between subgroups

Median (Q1 – Q3) StO_2_ was 65% (49 – 76) in OHCA patients, compared to 68% (64 – 74) in the shock group and 73% (67 – 79) in the ACS cohort, resulting in a significant overall difference (p < 0.01; Figure 2). Illustrating poorer regional perfusion in OHCA patient.

| **Comparison** | **mean difference** | **95% CI** | **p-value** |
| --- | --- | --- | --- |
| ACS vs. Shock | 3.7 | 2.8 – 4.5 | < 0.01 |
| ACS vs. OHCA | 9.8 | 8.1 – 11.5 | < 0.01 |
| Shock vs. OHCA | 6.2 | 5.6 – 7.7 | < 0.01 |

*Abbreviations: CI: confidence interval; ACS: acute coronary syndrome ; OHCA: out-of-hospital cardiac arrest*

# Supplement Table 4 - StO2 comparison with continuous and intermittent blood pressure measurements.

|  |  | **n** | **Pearson correlation** | **95% CI** |
| --- | --- | --- | --- | --- |
| **StO_2_** | Continuous SYS | 1420 | .172 | 0.121 to 0.222 |
|  | Continuous DIA | 1420 | .009 | -0.043 to 0.061 |
|  | Continuous MAP | 1420 | .125 | 0.073 to 0.175 |
|  | Intermittent SYS | 149 | .135 | -0.027 to 0.289 |
|  | Intermittent DIA | 149 | .075 | -0.087 to 0.233 |
|  | Intermittent MAP | 149 | .110 | -0.052 to 0.266 |

*Abbreviations: CI: confidence interval; SYS: systolic blood pressure; DIA: diastolic blood pressure; MAP: mean arterial pressure.*

# Supplement Table 5 - StO_2_ comparison in groups cMAP < 60mmHg and ≥ 60 mmHg for all patients

|  | **StO_2_ mean [%]** | **StO_2_ SD [%]** | **StO_2_ 95%CI [%]** | **StO_2_ n** | **p-value** |
| --- | --- | --- | --- | --- | --- |
| cMAP < 60mmHg | 67.8 | 11.7 | 66.1 – 69.5 | 189 | < 0.01 |
| cMAP ≥ 60mmHg | 71.6 | 7.9 | 71.2 – 72.1 | 1230 | < 0.01 |

*Abbreviations: CI: confidence interval; SD: standard deviation; cMAP: continuous mean arterial pressure; StO_2_: tissue oxygenation*

# Supplement Table 6 - StO_2_ comparison in groups cMAP < 60mmHg and ≥ 60 mmHg for patients in the “shock” group

|  | **StO_2_ mean [%]** | **StO_2_ SD [%]** | **StO_2_ n** |
| --- | --- | --- | --- |
| cMAP < 60mmHg | 72.9 | 6.1 | 140 |
| cMAP ≥ 60mmHg | 71.1 | 7.7 | 670 |

*Abbreviations: SD: standard deviation; cMAP: continuous mean arterial pressure; StO_2_: tissue oxygenation*

# Supplemental Figure 3 – Box Plots for StO_2_ comparisons


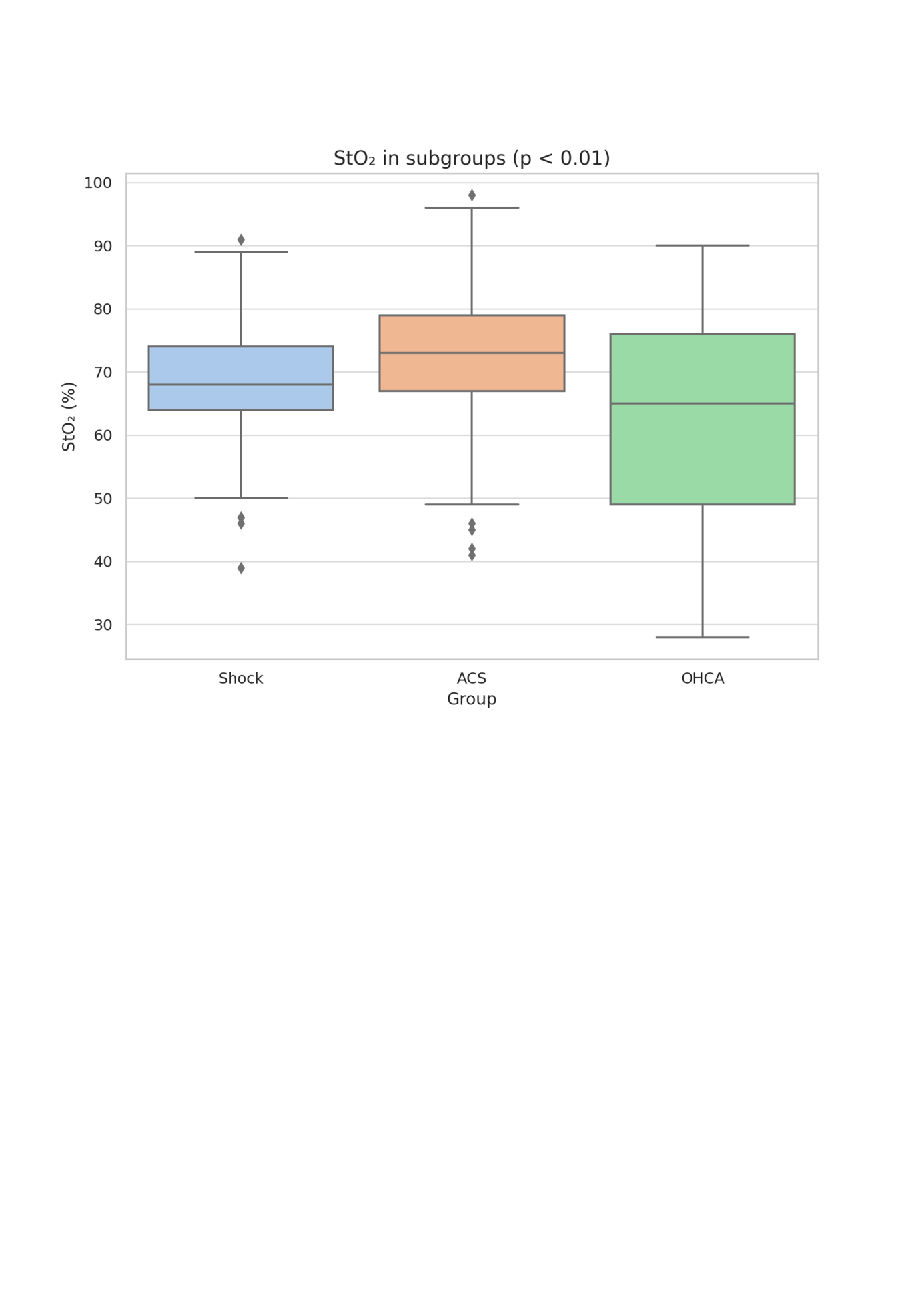


Boxplots of StO_2_ in each patient subgroup (Shock, ACS, OHCA). The central line represents the median, box IQR, and whiskers at the 95% confidence interval. Abbreviations: ACS: acute coronary syndrome; OHCA: out-of-hospital cardiac arrest

# Supplemental Figure 4 – Missed hypotension (MAP ≤60mmHg)


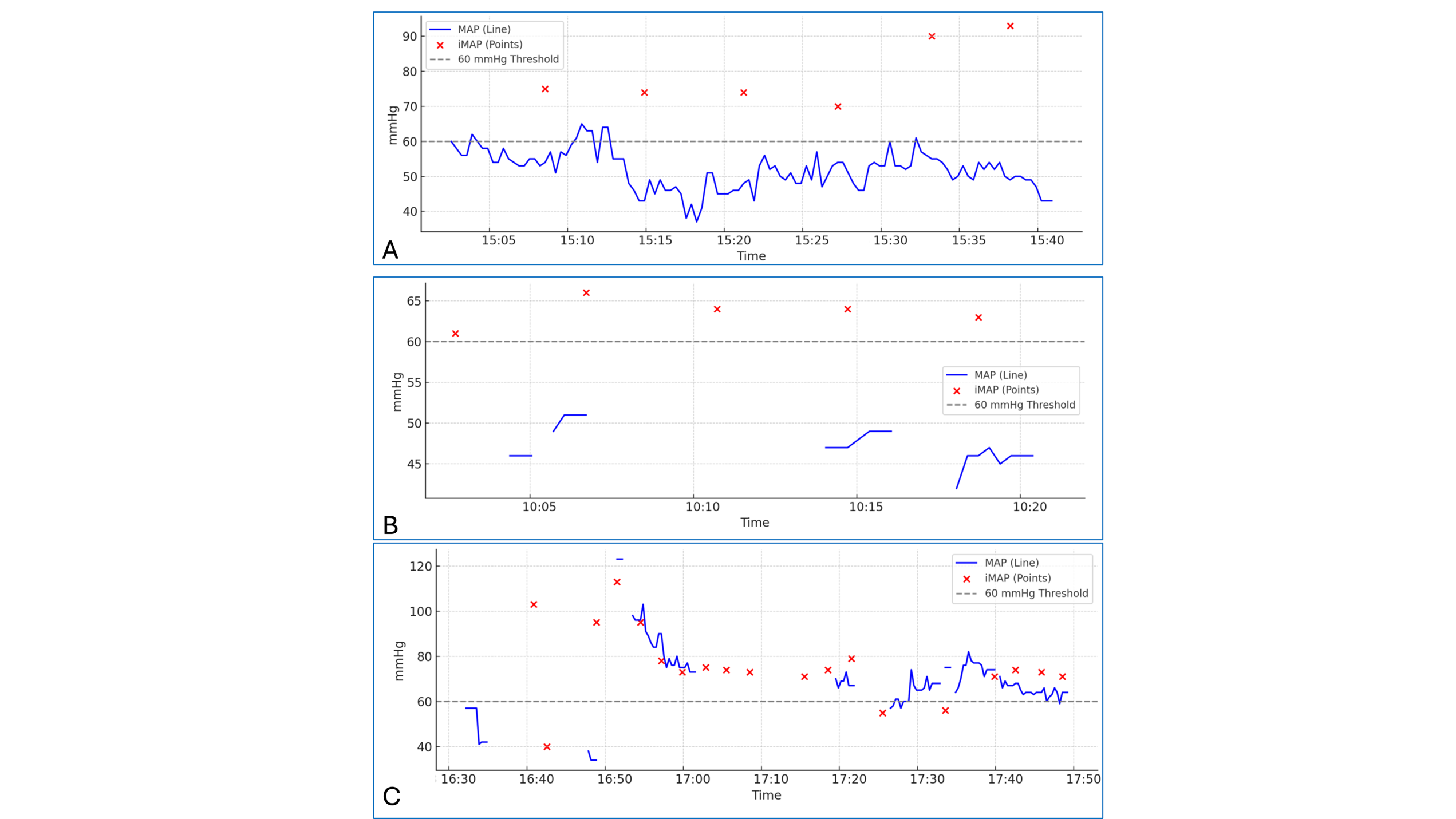


Abbreviations: iMAP = intermittent mean arterial pressure; MAP indicates continuously measured.

# References

1. Truijen, J., et al., *Noninvasive continuous hemodynamic monitoring.* J Clin Monit Comput, 2012. **26**(4): p. 267-78.

2. Boehmer, R.D., *Continuous, real-time, noninvasive monitor of blood pressure: Penaz methodology applied to the finger.* J Clin Monit, 1987. **3**(4): p. 282-7.

3. Eeftinck Schattenkerk, D.W., et al., *Nexfin noninvasive continuous blood pressure validated against Riva-Rocci/Korotkoff.* Am J Hypertens, 2009. **22**(4): p. 378-83.

4. Garnier, R.P., et al., *Level of agreement between Nexfin non-invasive arterial pressure with invasive arterial pressure measurements in children.* Br J Anaesth, 2012. **109**(4): p. 609-15.

5. Boushel, R., et al., *Monitoring tissue oxygen availability with near infrared spectroscopy (NIRS) in health and disease.* Scandinavian Journal of Medicine & Science in Sports, 2001. **11**(4): p. 213-222.

6. Benni, P.B., et al., *A validation method for near-infrared spectroscopy based tissue oximeters for cerebral and somatic tissue oxygen saturation measurements.* J Clin Monit Comput, 2018. **32**(2): p. 269-284.

7. Schober, P., C. Boer, and L.A. Schwarte, *Correlation Coefficients: Appropriate Use and Interpretation.* Anesthesia & Analgesia, 2018. **126**(5): p. 1763-1768.
